# The Postsurgical Clavien–Dindo Classification in Minor Surgery Can Improve Perception and Communication (Investigation on Blepharoplasty)

**DOI:** 10.3390/jpm12111900

**Published:** 2022-11-14

**Authors:** Gertraud Eylert, Christina Wolfsberger, Frederike Reischies-Meikl, Raimund Winter, Susan Dong, Birgit Michelitsch, Lars-Peter Kamolz, David Benjamin Lumenta

**Affiliations:** 1 Division of Plastic, Aesthetic and Reconstructive Surgery, Department of Surgery, Medical University of Graz, 8036 Graz, Austria; 2 Institute of Medical Science, School of Graduate Studies, University of Toronto, Toronto, ON M5S, Canada; 3 Plastic and Hand Surgery, University Hospital of Zurich, 8091 Zurich, Switzerland; 4 Division of Neonatology, Department of Pediatrics and Adolescent Medicine, Medical University of Graz, 8036 Graz, Austria; 5 Section of Infectious Diseases and Tropical Medicine, Department of Internal Medicine, Joint Facilities, Medical University of Graz, 8036 Graz, Austria; 6 Coremed—Centre for Regenerative Medicine, Joanneum Research Forschungsgesellschaft mbH, 8010 Graz, Austria

**Keywords:** blepharoplasty, complication, Clavien–Dindo Classification, satisfaction, perception, aesthetic cosmetic facial plastic reconstructive surgery

## Abstract

**The postsurgical Clavien–Dindo classification in minor surgery can improve perception and communication (Investigation on Blepharoplasty). Background:** Minor surgery lacks a standardized postoperative complication classification. This leads to the presentation of inaccurate postsurgical complication rates and makes comparisons challenging, especially for patients seeking information. This study aims to evaluate a standardized five-step complication grading system (Clavien–Dindo Classification, CDC) on the example of blepharoplasty, which is the most performed minor aesthetic surgery worldwide. **Methods:** A retrospective observational exploratory study of patients (N = 344) who received a bilateral upper eyelid blepharoplasty under local anesthesia from the same surgical staff was performed. Data were retrieved from the electronic patient record: the CDC grading and the surgeon-reported complications (N = 128) at the first follow-up on day 7. In addition, a telephone survey with patients (N = 261) after 6 months was performed, which consisted of 7 complication-related yes/no questions. **Results:** Based on the CDC, 41.6% of patients were classified as having no complications, and 58.4% had one. Furthermore, 1 patient (0.3%) received a revision under general anesthesia (CDC IIIb), 18 patients (5.2%) were re-operated under local anesthesia (CDC IIIa), 23 patients (6.7%) required pharmacological intervention (CDC II), and 159 patients (46.2%) had a complication from the normal postoperative course and received supportive treatment (CDC I). Moreover, 90.5% of the mentioned complications accounted for Grade I and II; 94% of the patients subjectively experienced no complications; 51% of patients were pleased with the surgery even though a complication occurred according to the CDC; 34% of complications escaped the awareness of the surgeon. **Conclusions:** Grade I and II complications occurred frequently. Complications escaped the perception of the patients and surgeons. The classification identifies a wide variety of postsurgical complications and allows a standardized comparison in minor surgery objectively. **Potential:** The CDC in minor procedures can improve the (institutional) preoperative communication with patients regarding potential postoperative expectations. Furthermore, the classification can be a useful tool to detect complication-related costs, identify insurance-related requests, and support evidence in medicolegal disputes. The example of blepharoplasty can be translated to various other and even less invasive procedures.

## 1. Introduction, Background

A blepharoplasty (“eyelid lift”) is the third most performed aesthetic surgery (after breast augmentation and liposuction) and the most performed minor aesthetic procedure under local anesthesia worldwide. It was performed more than 1.2 million times in 2019, [1] and can be performed by various physicians, including ophthalmologists, otolaryngologists, cranio-maxillofacial surgeons, dermatologists, general surgeons, and plastic and reconstructive surgeons. Blepharoplasty is a quick, minor, general elective, and is recognized as a safe procedure [2,3] to correct redundant skin and subcutaneous tissue due to the loosening of skin elasticity [4]. The procedure helps to improve the patient`s function again by increasing the field of vision, and aesthetically to enhance the quality of life with a rejuvenated facial appearance [5]. 

However, this popular surgery and other minor procedures lack an accurate standardized uniform postoperative complication classification system [5,6,7,8]. Specifically, complications in blepharoplasty are described as rare events [9]. For example, a single report described severe complications, such as retrobulbar hematoma in 0.05% with the most drastic complication being vision loss in 0.0045% of cases [9]. Furthermore, in the combination of blepharoplasty using an ablative carbon dioxide laser, complications are experienced (e.g., dermatitis or ectropium) [10], but are not adequately reported or perceived as complications at all. Comparable complication numbers are missing in the literature. In addition, different (institutional) scales and definitions are used to describe complication rates [5,6]. Due to this inconstancy, even meta-analyses of upper eyelid surgery could not be performed [6]. For the patient, this lack of comparative and ubiquitous definition of a postoperative complication is challenging, especially when seeking (aesthetic, cosmetic) information about surgery on the Internet [11,12,13,14] and social media [15,16,17]. Finally, the lack of a clear presentation of postoperative complications is a significant disadvantage for patient safety and informed consent [18,19,20,21]. It is necessary that the patient understands the procedure and is aware of postoperative expectations accordingly, especially in minor and aesthetic procedures. 

This study aimed to classify postoperative complications in blepharoplasty by applying the Clavien–Dindo Classification (CDC), a five-step postoperative classification scale (modified and reintroduced in 2004) [22,23]. The scale is based on interventions for postoperative complications, aiming to rank objectively in a standardized and reproducible manner. The CDC has even been adopted along with the Comprehensive Complication Index (in 2013). This allows a quantification of the overall burden of postoperative complications [24]. Although the CDC has been established as a standard in major and invasive surgeries [23,25,26,27,28], it has not been applied to minor, simple procedures performed under local anesthesia. In addition, the outcome was compared to the surgeon’s and the patient’s perceptions to analyze if the classification system corresponds with the general perception of postoperative complications after (this) minor intervention.

## 2. Material and Methods

### 2.1. Inclusion Criteria and Study Design

All adult patients (age > 18 years) who underwent a bilateral upper eyelid blepharoplasty under local anesthesia at a primary hospital in Austria (Division of Plastic, Aesthetic and Reconstructive Surgery, Department of Surgery, Medical University of Graz) between 2013 and 2016 were included (N = 344). All patients received their surgery from the same surgical team. Data from the electronic patient records (EPR) were collected and reviewed retrospectively by two independent reviewers and a qualitative observational study was conducted. 

### 2.2. Surgical Procedure and Follow-Up

**Figure 1:**
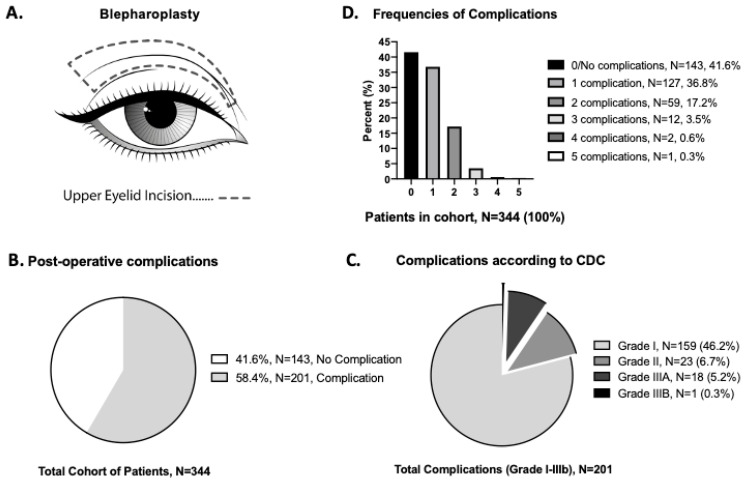
(**A**) Schematic upper eyelid blepharoplasty surgery, with an example of the surgical preoperative incision marking. (**B**) Result of postoperative complications if classified according to CDC. (**C**) Result of postoperative complications by CDC grading system (Grad I, II, IIIA, IIIB). (**D**) Result of frequencies of registered complications.

A blepharoplasty surgery was performed in our outpatient clinic. The operative time lasted from incision until the completion of the wound dressing. Most operations were performed using the same surgical technique: excision of skin with a scalpel blade 15+, orbicularis muscle strip resection +/− partial fat pad removal where appropriate, hemostasis during all steps with bipolar electrocautery, wound closure with 5.0 or 6.0 non-absorbable intradermal running stitch, steri-strip (skin color, 3M^TM^), which was performed in an outpatient setting (Figure 1A). Antibiotics were not routinely given for this procedure. The patient stayed several hours in our clinic for postoperative observation and was discharged the same day. In case the surgeon wanted to see the patient on the first postoperative day, an appointment was scheduled. However, all patients were routinely scheduled for a follow-up appointment on day 7 by the operating surgeon. A final follow-up took place six months after surgery if deemed necessary. 

### 2.3. Qualitative Assessment of Postoperative Complications

The primary outcome were postoperative complications, which were ranked according to the standardized CDC (Table 1) (available online: www.assessurgery.com (accessed on 17 October 2022)). As a Grade I complication, any deviation from the normal postoperative course without the need for pharmacological treatment or surgical, endoscopic, and radiological interventions is defined. Grade II is defined if pharmacological treatment with drugs other than those allowed for grade I complications is needed, including blood transfusions and total parenteral nutrition. Grad III is applied if any surgical intervention is required with the suffix “a” for all interventions under local anesthesia and the suffix “b” for all operations under general anesthesia. For further details see our referenced publication (Dindo D, et al., 2004) [22]. 

For the investigation on blepharoplasty surgery, for the readers of this manuscript, we added and highlighted in gray specific treatments in this minor procedure for visualization (e.g., lymphatic massaging/drainage or cooling for the treatment of swelling, or heparin ointments for the treatment of bruising/hematoma) (in light gray color, Table 1). 

### 2.4. Assessment of the Patient-Reported Complication Outcome

**Figure 2:**
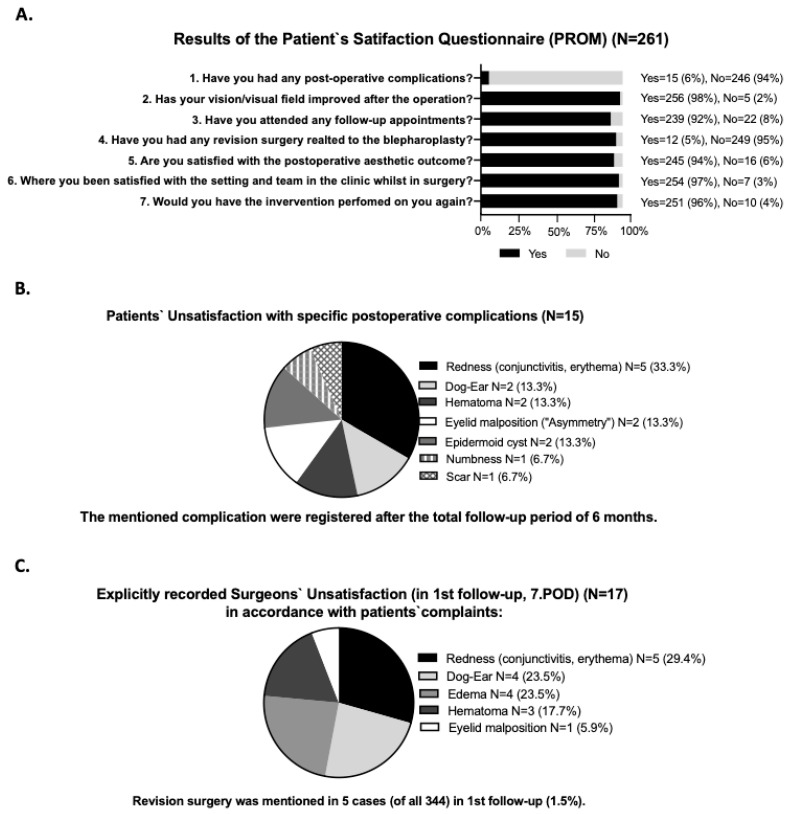
(**A**) Results of the patient`s satisfaction questionnaire (using a patient-reported outcome measure (PROM) survey questionnaire). (**B**) Result of the patients unsatisfaction with specific complications. (**C**) Result of explicitly recorded surgeons’ unsatisfaction on day 7, which were extracted from the patients who complained about any postoperative events after the surgery stated in the first follow-up to the surgeons.

Postoperative patient satisfaction and perception were evaluated at the earliest 6 months after surgery in 2017 via a complication-related telephone interview. The subjective survey was conducted by a single employee unaware of the patient`s medical history. Each patient was contacted a maximum of three times over different weeks of data collection and asked seven complication-related yes or no questions (as seen in Figure 2A). A total of 261 patients answered the telephone survey. The following items were polled: the occurrence of postoperative complications; satisfaction with postoperative visual field; further follow-ups, if any; revision surgery; satisfaction with the aesthetical outcome; satisfaction with the surgical team/setting in the clinic; if they would do the blepharoplasty surgery again. 

### 2.5. Assessment of the Surgeon-Reported Complication, Satisfaction, and Perception

The postoperative follow-up notes from the EPR were reviewed for subjective linguistic wording of the surgical outcome from the surgeons, such as “satisfying” (=positive) vs. “unsatisfying” (=negative), where a positive evaluation consisted of “favorable”, “appealing”, “happy”, and “good result”, and negatively interpreted wordings were “unsatisfied”, “revision surgery”, “unhappy”, and “unsatisfying result”. 

### 2.6. Comparison of the CDC vs. the Patients’ and Surgeons’ Satisfaction and Perception

In the case where data were available from the CDC and both the surgeon and the patient, the data were further matched for (dis-)agreement on the perception of a complication. In both scenarios, the assessment was made if a complication occurred, to evaluate the (dis-)agreement on the postoperative perception. Data were illustrated as descriptive cubes showing the percent of (dis-)agreement.

### 2.7. Statistical Analysis and Ethical Approval

Data were descriptively summarized and analyzed using Microsoft Excel (Microsoft^®^ Excel 2016 for Mac, Redmond, WA, USA). Data were anonymized and statistically analyzed in SPSS (IBM SPSS Statistics, Chicago, IL, USA, Version 20 for Mac). Data were expressed using means and standard deviation (SD), and the graphs were created with Prism (GraphPad Prism, San Diego, CA, USA, Version 8.0 for Mac). 

The institutional ethical review board of the Medical University of Graz, Austria approved the study protocol (EK-Nr. 29-396), and informed consent was obtained from all participants. 

## 3. Results

### 3.1. Patient Cohort Characteristics

A total of 344 patients (N = 344, 100%) who underwent a bilateral upper eyelid blepharoplasty procedure under local anesthesia were identified from the institution`s electronic patient records and evaluated (see Supplementary Material Table S1 for details).

### 3.2. Outcome Analysis of Blepharoplasty According to Clavien–Dindo

Of the 344 patients in the cohort study, 143 patients (41.6%) did not have any complications during the normal postoperative course. The other 201 patients had the following CDC documented complications (58.4%): 159 patients (46.2%) had a Grade I complication, where supportive treatment (e.g., topical treatment) was applied without further pharmacological or surgical intervention; 23 patients (6.7%) had a Grade II complication, which required a pharmacological intervention; 18 patients (5.2%) had a Grade IIIa complication, which required a surgical intervention without general anesthesia (such as hematoma evacuation, removal of an epidermal cyst, or tarsorrhaphy); 1 patient (0.3%) had a Grade IIIb complication, which required surgery under general anesthesia (Figure 1B,C). For a summary of the grade outcomes according to the CDC, see Table 2 for details.

When multiple complications were reported, the worst (highest Grade) complication was used for analysis (Figure 1C), and frequencies were reported (Figure 1D). According to the CDC, in 127 patients (36.8%), one complication was recorded; in 59 patients (17.2%), two complications were recorded; three complications occurred in 12 patients (3.5%); four complications occurred in 2 patients (0.6%); in 1 patient, five complications were recorded in the postoperative course (Figure 1D) (see Table 2 for details).

Interpretation:

- This table provides an overview of the complications that were recorded from patients in the data set (N = 344). It shows that 143 patients had no complications and 201 patients had complications.
- The first row (Hematoma) shows 143 patients with no (hematoma) complications and 54 patients with a hematoma complication (last column). Of these 54 patients, 45 patients were classified as a Grade 1 complication, 4 patients were classified as a Grade 2 complication, etc.
- The gray-shaded color palette refers to the complication grading (as seen in Figure 1B,C).

### 3.3. Patients’ Satisfaction and Perception of the Complication-Related Telephone Survey

The patient satisfaction and perception scores were assessed by a telephone interview after a patient had completed all follow-ups. A total of 261 patients participated (75.9%). From all interview participants, 208 patients (79.6%) were female (response ratio 0.8) and 53 patients (20.4%) were male (response ratio 0.7).

Most of the 246 patients (94%) subjectively experienced no complication, whereas 15 patients (6%) reported having one. Additional results obtained were that 256 patients (98%) were satisfied with the regained field of vision, compared to 5 (2%) who were not; 239 patients (92%) stated they went to the first clinical follow-up appointment after surgery, and 22 (8%) did not attend any follow-ups; 12 patients reported having undergone revision surgery (5%), of which ten were performed at our clinic and the other two elsewhere; 245 patients (94%) were satisfied with the aesthetic outcome, and 16 patients (6%) said they were not. In the following question, our interviewer evaluated the satisfaction with the surgical team/setting in the clinic and received positive feedback from 254 patients (97%) vs. negative feedback from 7 patients (3%). The final key question polled whether the patient would do the surgery again, and 251 patients answered with “yes” (96%) compared to 10 patients (4%) who answered “no” (Figure 2A). 

### 3.4. Extraction of Patients’ Satisfaction of Complications

Fifteen patients stated they had, according to their perception, a complication. Here, the patients were further evaluated, and the complications were extracted from the data set. The following complications were perceived as complications: redness, such as conjunctivitis and erythema in five cases (33.3%), dog-ears in two patients (13.3%), hematoma in two patients (13.3%), an eyelid malposition with an asymmetry in two patients (13.3%), an epidermoid cyst in two patients (13.3%), a feeling of numbness in one patient (6.7%), and a scar in one patient (6.7%). (Figure 2B). 

**Figure 3:**
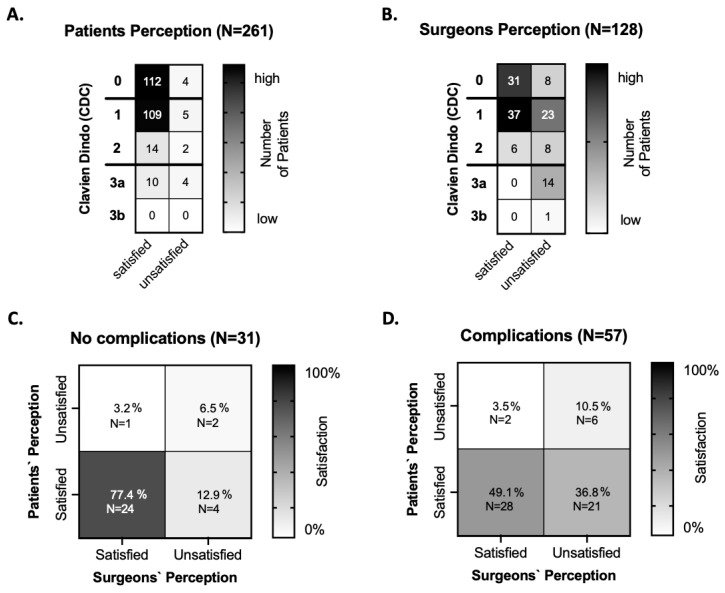
(**A**,**B**) Result of patients’ and surgeons’ perception of the CD classified complications with stated (un-)satisfaction. Horizontal black thick lines in Graph (**A**,**B**) divided as followed: 0 (no complication), 1 and 2 (“mild/minor” complication) (Grad I and II), where conservative treatment was applied, and 3a and 3b (“major” complication) (Grad IIIa, IIIb), where a revision surgery was performed under local and general anesthesia. The color scale (vertically) in all **Graphs** (**A**–**D**) on the right side of the Graphs indicates the amount of the number of patients. (**C**,**D**) Results of the comparison of matching (dis-)agreements from both the patients’ (*y*-axis) and surgeons’ statements (*x*-axis) from the EPR, in case no complications occurred (**C**) and in case complications were classified (**D**).

In an analysis of the perception and the correlated graded CDC, one hundred twenty-three patients were satisfied with the postsurgical outcome despite a complication occurring and a supportive (N = 109) or a pharmacological (N = 14) treatment being applied. In addition, ten patients were satisfied even though revision surgery was needed. In total, one hundred thirty-three patients (109 + 14 + 10, 51%) were pleased with the surgery even though a complication occurred according to the CDC. Four patients were unsatisfied with the surgery caused by the set-up and the overall unsatisfaction with the surgical team, the clinic, and long-waiting times in the outpatient clinic (Figure 3A).

### 3.5. Outcome of Surgeons-Related Complications Regarding Satisfaction and Perception

On day 7 in this analysis, 128 records were analyzed, which contained a specific subjective statement (positive/negative wording) from the surgeon regarding perception (37.2% of N = 344). The surgeons stated, in 74 cases (21.5% of N = 344), a surgical result they were satisfied with, and in 54 patients (15.7% of N = 344), they were unsatisfied with the postsurgical outcome. In further analysis, the perception was correlated to the graded CDC. Furthermore, 43 surgeons (37 + 6) were satisfied even when a complication Grade I and II occurred, representing 34% of complications that escaped the awareness of the surgeon. In 8 cases, the surgeon used negative linguistic wording in the perception analysis without mentioning the exact reason (Figure 3B).

The surgeons were explicitly “unsatisfied” with the following five postoperative complications: redness, such as conjunctivitis and erythema in 5 cases (29.4%), dog-ears in 4 cases (23.5%), edema in 4 patients (23.5%), hematoma in 3 patients (17.7%), and eyelid malposition in 1 patient (5.9%). In 5 patients (1.5%) the surgeons discussed revision surgery during the initial follow-up as documented in the medical record (Figure 2C).

In the follow-up, six months after the surgery 112 records (32.6% of N = 344) contained a statement regarding perception. All surgeons were satisfied with the “aesthetical/cosmetical result”, as well as in cases where revision surgery was performed. No further follow-ups were scheduled.

### 3.6. Comparison of Patients’ and Surgeons’ Perception

An assessment of the awareness of postoperative complications was made by patients and surgeons on the first follow-up on day 7. The perception was then compared to the scored CDC. On day 7, 261 patients’ and 128 surgeons’ opinions were assessed. 

Even though complications occurred, the patients and the surgeons were very satisfied (109 of 261) and did not recognize the complication as such. In addition, more than two-thirds of the patients were also satisfied even though a minor revision was needed (10 vs. 4 patients). Surgeons were more critical and were all unsatisfied when the complication required a revision (15 of 15 surgeons) (Figure 3A,B).

The records were further matched when a subjective statement was available in the same EPR from both the patient and the surgeon. When a complication did occur, 77.4% agreement was recorded that the patient and surgeon stated a “satisfying outcome”, while 22.6% had an “unsatisfying outcome”. In the case where a complication according to the CDC (Grade I, II, IIIa, and IIIb) was recorded, there was still 49.1% satisfaction reported, with 50.8% unsatisfied. Surgeons were more unsatisfied (36.8%) with a postoperative complication compared to 3.5% of patients (Figure 3C,D).

## 4. Discussion

Collecting surgical data is key for assessing and improving the quality and safety of a procedure. The CDC provides a standardized tool to describe postoperative complications that are based on interventions (follow-up treatments). In this study, we showed that the CDC can be used to evaluate minor surgeries by identifying a wide variety of potential postsurgical risks and complications (evaluated on the first follow-ups on day 7) that required therapy. The classification system provides an objective tool for surgeons to analyze institutional practice and, therefore, improves the preoperative communication with patients for informed consent, identifying potential interventions, which are neither by the patients nor the surgeons perceived as complications as such. This investigation showed, surprisingly, that in almost 60% of all performed surgeries, a therapy was initiated by the surgeon, and that 90.5% of the complications were “mild” Grade I and II where conservative treatment (supportive and pharmacological treatment) was prescribed. The CDC identified lots of complications in this minor procedure and, therefore, fills a valuable gap in describing the postsurgical results and provides necessary information on postsurgical expectations for the patients. No Grade IV and V complications occurred, confirming that the procedure is safe. The CDC, as shown in the example of blepharoplasty, allows translation to various other disciplines and even less invasive procedures (such as fillers, injections, laser, and medical tattooing), which lack the identification and quantification of complications (such as disturbing skin hyperemia, infections, necrosis, burns). By identifying postsurgical complications, it is possible to implement the appropriate prevention measures. 

There is no consensus on when the CDC should or can be applied. We chose the first follow-up in this generally elective procedure conducted in our outpatient clinic. The retrieved surgical data helped our institution to implement postsurgical precautions, such as the standardized use of a “cooling period” after the procedure. Consequently, we found reduced complaints about swelling in the first follow-up (no data shown here). 

For a detailed discussion of our key findings of various complications in blepharoplasty and interpretation see Supplementary Material Table S2.

By using the CDC for classifying postsurgical complications, it must be highlighted that a classified complication is not synonymous with a medical error, as seen in the example of swelling and bruising, which are also associated with a natural consequence of any surgery (wound healing phases: hemostasis, e.g., bleeding, inflammation, e.g., swelling). The CDC can help the surgeon guide patients’ postoperative expectations and initiate preventive information and treatment. Patients must understand the purpose of a reintervention. Since our investigation, we have implemented the CDC routinely in our clinic for minor procedures. We improved our informed consent and, hence, subjectively, we received positive feedback from our patients. 

Finally, further investigations and reports are needed to establish the CDC for minor procedures. Respectively, other authors need to confirm that the CDC is a reliable scale in their institutions. However, further implementation in minor procedures could add important information to the literature and online information on the Internet for patients. Our investigation can be easily translated to any other minor (surgical) procedure. 

## 5. Limitations

The first follow-up was scheduled 7 days after the surgery and 58.4% of complications were observed according to the CDC. It is possible that this is not sufficient time to observe all complications; however, the most frequent complications, such as hematoma and infections, could be detected as well as the worst complications, including a retrobulbar hematoma. Scarring, as sequelae beyond an observation period of 6 months, were not included in this analysis. 

The study population was possibly biased toward greater satisfaction in the qualitative research analysis. Most blepharoplasties were covered by public health care insurance (approval criteria: resectable excess of the upper eyelid skin and the presentation of an objective report confirming a cranio-temporal reduction in the bilateral visual field). 

Multiple other sophisticated PROMs (e.g., FACE-Q [29,30]) are available; however, they were not suitable (not standardized) for our quick and straightforward telephone-based poll; we implemented a control and key question to account for this limitation (“if the patient would do the surgery again”). We recorded objective visual complications documented via photography. In the telephone interview, we also recorded the complication of “numbness”, which was not detected earlier (without any further medical intervention). Since this was a retrospective analysis, just 37.2% of subjective statements from the surgeons regarding perception could be included, which were analyzed compared to 75.9% of subjective statements from the patients on day 7. This was most likely due to the volume of patients in the outpatient clinic and the short notes after a consultation. In addition, we lost patients in the personal follow-up, which was most often caused by the pleasing functional and aesthetic postoperative result. Many patients did not want to come back to our outpatient clinic because their medical problem was solved. However, via telephone survey, we were able to include 75.8% of the patients in our investigation.

## 6. Future Directions and Recommendations

The CDC is applicable for minor surgical interventions in the immediate postoperative period. Modifications should be made to account for the inclusion of long-term complications, which might be carried out by adding a letter, such as the suffix “L” indicating “late complication(s)” (LCI) or “S” as sequelae (SCI), for example (see Table 1, suffix “d” for disability).

Furthermore, we suggest bearing “supportive measures” in mind when classifying Grade I complications (“any aberration from the normal postoperative course”), with examples being lymphatic massaging as well as supporting creams and ointments for reducing swelling. Furthermore, we specifically added the word “antibiotics” to Grade II (orally or parenteral) because we believe antibiotics are given with the intention-to-treat (which are absorbed into the bloodstream) rather than as a (topical) support. 

After this assessment, our institution included in our routine the application of “cooling” (gel) eyeglasses after surgery for 15 min for each patient. So far, we have observed a positive outcome; however, a follow-up assessment utilizing the CDC has not been performed. Other authors, for instance, recommend avoiding any blood-thinning medications ten days before surgery [31] or recommend lymphatic drainage before surgery, which we consider in the informed consent.

Ultimately, a standardized, reproducible classification could help further develop and calculate perioperative individual risk calculations. 

## 7. Conclusions

Based on our evaluation, we successfully applied the CDC in a generally safe, minor elective surgical procedure under local anesthesia and support its use for future clinical practice and research. This investigation attempted to use a standard framework that can guide the awareness of postoperative complications. Institutionally generated data from the CDC can be used to improve (institutional) informed consent before surgery and provide more information on postoperative expectations and implement quality and preventive safety measures for procedures. Postoperative comparisons would be objectively possible. Furthermore, the implementation of the CDC would help both the patient and the surgeon when it comes to expected costs for reinterventions and necessary follow-ups, questions from insurance companies, help in legal disputes, and, ultimately, can improve the immediate expectations after surgery.

## Figures and Tables

**Table 1:** Clavien–Dindo Classification ^§^ modified for Blepharoplasty (light grey color).

Grade	Definition
Grade I	Any deviations from the normal postoperative course without the need for pharmacological treatments or surgical interventions, although therapy allowed: antiemetics, antipyretics, analgesics, diuretics, electrolytes, skin repair creams, heparin ointments, moisturizing eye drops. Additionally included are wound infections opened at the bedside. Additional conservative therapy, such as lymphatic massaging/drainage and cooling.
Grade II	Requiring pharmacological treatment with drugs (such as antibiotics) other than for grade I complications, such as blood transfusions and total parenteral nutrition.
Grade III	Requiring surgical intervention.
Grade IIIA	Under local anesthesia.
Grade IIIB	Under general anesthesia.
Grade IV	Life-threatening complication (including CNS complications) * requiring IC/ICU.
Grade IVA	Single organ failure (including dialysis).
Grade IVB	Multiorgan failure (MOF).
Grade V	Death due to the intervention.
Suffix “d”	If the patient suffers from a complication at the time of discharge, the suffix “d” (for “disability”) is added to the respective grade of complication. Follow-up is required.

* Brain hemorrhage, ischemic stroke, subarachnoidal bleeding, but excluding transient ischemic attacks. CNS, central nervous system; IC, intermediate care; ICU, intensive care unit. *(In grey, modifications for blepharoplasty) *^§^: Dindo D, Demartines N, Clavien P-A. Classification of Surgical Complications. Ann Surg 2004;240:205–213.

**Table 2:** Outcome complications according to CDC Grading.

Complications		No Complication	Grade 1	Grade 2	Grade 3a	Grade 3b	Total N = 344
Hematoma	**no**	143	114	19	14	0	290
(Ecchymosis)	**yes**	-	45	4	4	1	54
Edema	**no**	143	34	11	9	0	197
(Swelling)	**yes**	-	125	12	9	1	147
Redness (conjunctivitis, erythema)	**no**	143	155	4	17	1	320
	**yes**	-	4	19	1	0	24
Chemosis	**no**	143	142	19	18	1	323
	**yes**	-	17	4	0	0	21
Wound dehiscence	**no**	143	148	20	16	1	328
	**yes**	-	11	3	2	0	16
Eyelid malposition (ectropion, wrinkle)	**no** **yes**	143 -	157 2	23 0	17 1	1 0	341 3
Inocclusion	**no**	143	158	22	16	0	339
	**yes**	-	1	1	2	1	5
Eye ptosis	**no**	143	156	23	15	1	338
	**yes**	-	3	0	3	0	6
Eye deviation	**no**	143	159	23	17	1	343
	**yes**	-	0	0	1	0	1
Fat hernia	**no**	143	159	23	17	1	343
	**yes**	-	0	0	1	0	1
Epidermoid cyst	**no**	143	159	23	13	0	338
	**yes**	-	0	0	5	1	6
Dog-ear	**no**	143	159	23	15	1	341
	**yes**	-	0	0	3	0	3
Retrobulbar	**no**	143	159	23	17	1	343
hematoma	**yes**	-	0	0	1	0	1

## Data Availability

The datasets generated and analyzed in this work are available from the corresponding authors upon reasonable request.

## Supplementary Materials

The following supporting information can be downloaded at: https://www.mdpi.com/article/10.3390/jpm12111900/s1, Table S1 Patient Cohort Characteristics; Table S2 Detailed Discussion of Key Findings [32,33,34,35,36,37,38].

## Abbreviations

CDC	Clavien–Dindo Classification
EPR	electronic patient records
PROM	patient-reported outcome measure
SD	standard deviation
