# Supplementary material for: The Postsurgical Clavien–Dindo Classification in Minor Surgery Can Improve Perception and Communication (Investigation on Blepharoplasty)
Source: J Pers Med. 2022 Nov 14;12(11):1900. doi: 10.3390/jpm12111900 (PMC9692875; doi:10.3390/jpm12111900)
Supplement: Supplementary file 1 [file jpm-12-01900-s001.zip › jpm-2005994-supplementary.pdf]

SUPPLEMENTARY MATERIAL

Supplementary Material Table S1. Patient`s Cohort Characteristics of Blepharoplasty Surgery

Patient`s Cohort Characteristics of Blepharoplasty Surgery			
	**Number (N)**	**Percent (%)**	**Mean (range)**
**Bilateral upper lid blepharoplasty under local anesthesia**	344	100%	
**No antibiotics during procedure**	344	100%	
**Patients**	344	100	
female	278	80.8	
male	66	19.2	
**Age (years)**			57 (21 to 90)
**BMI (kg/m²)**			26.4 (SD,36.7)
**Smoking**			
no	273	79.3	
yes	71	20.7	
**Allergies**			
no stated allergy	318	92.4%	
antibiotics (penicillin)	18	5.2%	
surgical gloves	4	1.2%	
wound dressing (plaster-patches)	3	0.9%	
non-steroidal anti-inflamm. drug (NSAIDs)	1	0.3%	
**Duration (min)***			52 (33 to 157)
**Additional Surgery (in the same setting)**			
excision of a naevi (face)	7	2.0	
eyebrow lift (bilateral)	6	1.7	
excision of a tumor (eyelid)	3	0.9	
eyebrow lift (unilateral)	2	0.6	
excision of a lipoma (forehead)	1	0.3	
extirpation of xanthelasma	1	0.3%	
trigger finger (hand surgery)	1	0.3%	

*start with first cut until completion of the wound dressing

Supplementary Material Table S2. Key Findings and Discussion

**Complication rates**	• We found no Grade IV and V complications, confirming that **blepharoplasty is a safe procedure**.<br>• **Revisions** were performed **in 5.5%**, with one (0.3%) that was performed under general anesthesia due to the explicit wish of the patient.<br>• **We identified an overall complication rate of 58.4%**. The majority were "mild/minor" complications (Grade I and II) that were self-resolving, which accounted for 90.5% of all complications, and were supported by conservative and pharmacological treatment. Other authors testing the CD classification system also found higher complication rates than expected, especially in the analysis of aesthetic plastic surgery (between 50-60%) [26,27,32].<br>• **We identified 90.5% of minor complications**, which were primarily self-resolving over time (edema/swelling=most frequent, hematoma/ecchymosis=second frequent, etc.); they were registered 7 days postoperatively – within the (immediate) surgical course. However, speculate that the "prolonged" edema, which was also the most frequent complication in a multi-center evaluation of >4,000 patients [6] occurred primarily due to the volume (and fluid overload) of the injected anesthesia, and specifically the hydro dissection (which is not usually mentioned in the Informed Consent).<br>• In contrast, **41.6% of patients did not have any signs of a postoperative complication** in our cohort (with absolutely no swelling and no bruising, just the in-situ suture material).<br>• From a surgical perspective, our findings are very interesting and necessitate the **discussion of what is considered normal symptoms of postoperative recovery**, such as edema/swelling or hematoma/ecchymosis.<br>• **The CDC also allowed the identification of 13 different complications on day 7**. Interestingly, other authors evaluating periorbital fat augmentation in >4,000 patients identified even 47 complications [6] after surgery (even with a longer follow-up time); including deep wrinkles, skin laxity, worsening of dark circles, melanosis, hyperpigmentation, or hollowness were also registered. This indicates that any deviations from the normal (or no postoperative changes) can be identified as a complication and further identified if treatment was needed and applied (e.g., conservative, pharmacological, or surgical).<br>• **Bleeding and hematoma**, which occurred frequently and resolved within 2-3 weeks, was the complication surgeons were mainly unsatisfied with. This highlights that physicians are more prone to mentioning a complication in medical records, specifically if a (major) visual appearance from the normal can be seen.

**Comparison of surgical outcome**	• **A ubiquitously used complication scale would help medical professionals** who even disagree on the definition of major (i.e., under general anesthesia, among others) and minor surgeries as recently discussed (2021) [8].<br>• **A universal and transparent complication scale would foster the doctors-patient relationship** and benefit both sides if postoperative complications occur. In addition, surgeons can openly discuss the non-surgical management, including the (institutional) rates of minor complications (CDC Grade I and II) before a planned procedure.<br>• Especially with the increasing trend in (minor) aesthetic surgery, **raising awareness of potential postoperative complications could improve coping with early complications and improve communication of what to expect after an operation**. This would help lead to unsatisfied patients regarding the postoperative prognosis, functional and aesthetic recovery time, and psychological management [33–35]. Especially, in times when patients seek (aesthetic, cosmetic) information on the internet [11,12,32], where expectations are high [21,36].<br>• We believe reporting on all potential complications, especially minor ones, such as swelling or hematoma is important to provide accurate information on the expected surgery for patients [36,37] and surgeons [38]. Therefore, an objective, reproducible complication rating scale would benefit from comparison.
**Perception and correlation with the CDC**	• In the analysis, **we found that the overall patient satisfaction was high** (with 98% satisfied with the field of vision and 94% satisfied aesthetically). **Only 6% subjectively experienced a medical complication postoperatively.** This is interesting since patients may be unaware of risks and complications and can be improperly informed when seeking surrogate information on the Internet and social media[15–17].<br>• Interestingly, these classified complications (CDC Grade I or II) are escaping the awareness of most patients (94%) and also the perception of the surgeons (34%).
